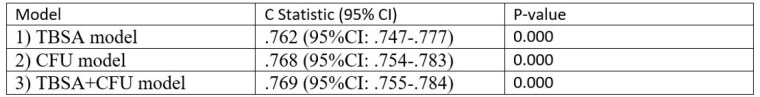# 28 Cutaneous Functional Units versus Total Body Surface Area– Which Is Better for Predicting Motion Loss?

**DOI:** 10.1093/jbcr/iraf019.028

**Published:** 2025-04-01

**Authors:** Ingrid Parry, Daniel Tancredi, Janice Bell

**Affiliations:** University of California, Davis and Shriners Children’s - Northern California; University of California Davis; University of California Davis

## Abstract

**Introduction:**

Total body surface area (TBSA) is a well-known risk factor for short-term outcomes after burn injury, such as mortality or sepsis. It is less effective for predicting longer-term outcomes like burn scar contractures. Early and reliable identification of patients at risk of contractures and other scar-related problems could help guide preventative efforts and improve outcomes for burn survivors. Cutaneous Functional Units (CFUs) offer a potential metric for predicting scar outcomes because they quantify burn severity and extent within more localized and functionally relevant areas of the body. This study compared predictive models using CFUs, TBSA, and both CFU+TBSA to determine the value of incorporating CFU data into models that predict loss of range of motion (ROM) after a burn injury.

**Methods:**

The following predictive models were compared: 1) TBSA - included patient-level extent (%) of burn; 2) CFU - included % of burn within each defined CFU area; and 3) CFU+TBSA - included % burn at both body-level and CFU-level. All models included core variables of sex, age, race, and individual joint motion. The outcome variable was ROM, measured at hospital discharge at the joint level using degrees converted to a proportion of typical ROM. Model discrimination was assessed using the Area Under the Receiver operating characteristic curve (Harrell’s C statistic). The models were developed using fractional regression and logit function with one dataset and validated on an independent dataset to ensure unbiased assessment.

**Results:**

Models were developed using data from a prospective, longitudinal, multi-site cohort study including 7463 joint motions from 307 adult burn survivors. The combined CFU+TBSA model had the highest C statistic, followed by the CFU model, then the TBSA model (Table 1). The improvements in the C-statistic of the CFU model (p=0.03) and TBSA+CFU model (p=0.00) were statistically significant compared to the TBSA model but were not different from each other (p=0.48), suggesting that models including CFU are better. The CFU +TBSA model was applied to an independent dataset of 66 patients and 1467 joint motion observations from a prospective cross-sectional study of adults with burn scar contractures. The C statistic was.639 (95%CI: 0.571, 0.707; p=0.00), demonstrating significant but lower discrimination in this external dataset.

**Conclusions:**

Predictive models that include CFUs alone or CFU+TBSA can better distinguish joint motions at risk for loss of ROM than TBSA alone. The CFU+TBSA model also demonstrated moderate discriminatory power in an independent dataset. This study established the statistical value and utility of using CFUs to predict motion loss and sets the stage for further investigation, including clinical relevance and validation with other patient samples.

**Applicability of Research to Practice:**

Prediction of scar problems after burn injury.

**Funding for the Study:**

N/A